# Evaluation of Trabecular Microstructure of Cancellous Bone Using Quarter-Detector Computed Tomography

**DOI:** 10.3390/diagnostics13071240

**Published:** 2023-03-25

**Authors:** Hiroaki Hasegawa, Nobuhito Nango, Masafumi Machida

**Affiliations:** 1 Department of Radiological Sciences, International University of Health and Welfare, Narita 286-8686, Japan; 2 Ratoc System Engineering Co., Ltd., Tokyo 162-0801, Japan; 3 Department of Orthopedic Surgery, Hakujikai Memorial Hospital, Tokyo 123-0864, Japan

**Keywords:** osteoporosis, quarter-detector computed tomography, micro-computed tomography, trabecular microstructure, cancellous bone

## Abstract

Quarter-detector computed tomography (QDCT) is an ultra-high-spatial-resolution imaging technique. This study aimed to verify the validity of trabecular structure evaluation using a QDCT scanner in the diagnosis of osteoporosis. We used a cancellous bone specimen image of the second lumbar vertebrae of an adult male with moderate osteoporosis. To obtain QDCT images, we created a three-dimensional model from micro-CT images of the specimen. Statistical analysis was performed on the relationship between micro-CT and QDCT imaging modalities. The differences between micro-CT and QDCT were assessed based on their significance with respect to the calculated mean measurements using the Mann–Whitney test. Single regression analysis was performed using linear regression, with micro-CT and QDCT as the explanatory and objective variables, respectively, to determine the relationship of the measured values between the two modalities. By applying the necessary correction to the micro-CT measured values, it is possible to perform an analysis equivalent to micro-CT, which offers higher spatial resolution than QDCT. We found evidence that if QDCT can be used, trabecular structure evaluation may contribute to image diagnosis to evaluate practical bone fragility.

## 1. Introduction

The technology of X-ray computed tomography in diagnostic imaging has made great progress in recent years. Ultra-high-resolution computed tomography, termed quarter-detector computed tomography (QDCT), is a type of X-ray multidetector-row CT (MDCT) that can acquire images with higher spatial resolution than conventional MDCT. The resolution in the slice direction achieves a minimum detector-row width of 0.25 mm compared to the conventional 5 mm width. Concerning the resolution within the slice plane, 512 matrices are generally used for CT images, but QDCT can acquire images reconstructed using 1024 or 2048 matrices. By reducing the detector width in the slice direction and increasing the number of image matrices, the spatial resolution becomes double that of conventional MDCT [1]. These techniques have been put to practical use in medical X-ray CT scanners by improving the accuracy of bed driving and reducing the focus of the X-ray tube. The QDCT scanner has made it possible to image fine structures of peripheral bronchi [2,3], microvasculature [4,5], and bone structures [6] by obtaining high-spatial-resolution images.

In the bone region, high-contrast images can be obtained by X-ray CT, and bone fragility assessment based on morphological structure analysis can be performed [7,8,9,10,11,12]. Trabecular morphology correlates with bone mineral density and is a factor in fracture susceptibility [13,14,15,16,17]. High-resolution peripheral quantitative computed tomography (HR-pQCT) [18,19,20] analyzes the trabecular morphology of cancellous bone, as opposed to bone mineral density or bone composition analysis obtained by dual-energy X-ray absorptiometry (DEXA). Quantitative CT is a non-invasive procedure intended for the human body, but it is only used for peripheral bones.

Bone fragility assessment using QDCT scanners is used for image diagnosis of breast and prostate cancer. Hormone therapy with tamoxifen and luteinizing hormone-releasing hormone (LHRH) agonists is recommended for endocrine therapy of breast cancer. Tamoxifen (an anti-estrogenic drug) reduces bone mineral density in premenopausal patients [21]. The same is true for LHRH agonists that suppress ovarian function [22], and chemotherapy also causes a loss of bone mineral density during artificial menopause [23]. Similarly, androgen deprivation, a recommended endocrine therapy in prostate cancer, also causes bone mineral loss [24,25]. Annual bone mineral scanning should be performed for secondary osteoporosis associated with breast and prostate cancer treatment [26,27]. Patients on hormone therapy require periodic CT scans to assess treatment efficacy, as well as quantitative bone mineral scanning. Fracture risk assessment for secondary osteoporosis influences treatment policy decisions and the patients’ quality of life. If bone fragility assessment, that is, with a diagnosis equivalent to quantitative CT, can be performed in whole-body CT scans during follow-up, it will be possible to reduce the physical burden and exposure dose of patients. The addition of bone strength information to whole-body CT scans is expected to be beneficial from the perspective of orthopedic imaging diagnosis and medical exposure reduction.

In this study, we aimed to verify the validity of trabecular structure evaluation using a QDCT scanner in the diagnosis of osteoporosis. Quantitative CT requires image analysis with high spatial resolution to understand the macroscopic morphological properties of the bones. It is common to use a micro-CT scanner for the analysis for animal experiments and bone analysis of specimens. For this reason, and to evaluate the clinical superiority of the QDCT scanner, verification with a micro-CT scanner is necessary. The purpose of this study was to determine the possibility of trabecular bone structure evaluation using QDCT, and to provide evidence for the deployment of QDCT in general-purpose image diagnosis to evaluate practical bone fragility.

## 2. Materials and Methods

### 2.1. CT Imaging

In this study, we used bone as our specimen and imaged the second lumbar vertebrae of an adult male. No personal information was included before the participant’s death. The lumbar vertebrae were provided by Nihon University School of Medicine, and their use was approved by the Nihon University School of Medicine Ethics Review Committee. Images of the specimen lumbar spine were acquired using a micro-CT scanner. The lumbar spine specimen demonstrated bore moderate osteoporosis, as evidenced by the denseness of the cancellous bone. The micro-CT scanner (TOSCANER-32250) manufactured by Toshiba IT Control Co., Ltd. (Tokyo, Japan) was used, and images were acquired using a 90 kV tube voltage and 25 μA current. The voxel dimension at the time of image acquisition was set at 0.05 mm. Next, to obtain QDCT images, a three-dimensional (3D) model was created from the micro-CT images. A ProJet MJP 3600 MAX 3D printer (3D SYSTEMS, Inc., Rock Hill, SC, USA) was used. A resolution of 757 × 750 × 1600 dots per inch and a lamination pitch of 16 μm were used. The modeling method was an inkjet method using an ultraviolet curable acrylic resin. Using the 3D model, transverse images were acquired with a medical X-ray CT scanner, i.e., an ultra-high-definition MDCT scanner (Aquilion Precision) manufactured by Toshiba Medical Systems Corporation (Otawara, Japan). A 3D model of the lumbar vertebrae was placed on the couch so that the craniocaudal direction and the transverse plane of the lumbar vertebrae coincided and were at the center of the scan field of view. The imaging conditions were as follows: 120 kV tube voltage, 50 mA tube current, and 0.5 s/rotation X-ray tube rotation speed. The X-ray tube focal size (according to IEC 60336:2005) was set to 0.4 × 0.5 mm, which is the smallest among the available focal sizes for QDCT. For detector configuration, 0.25 × 160 rows of helical scanning were used. A pitch factor of 0.806 was used to indicate the couch moving distance with respect to the X-ray beam. For image reconstruction, we used the function FC13 (for the body), the slice thickness was 0.25 mm, slice interval was 0.25 mm, the number of image matrices was 1024, and the reconstruction field of view was 160 mm.

### 2.2. Bone Morphometry

**Figure 1:**
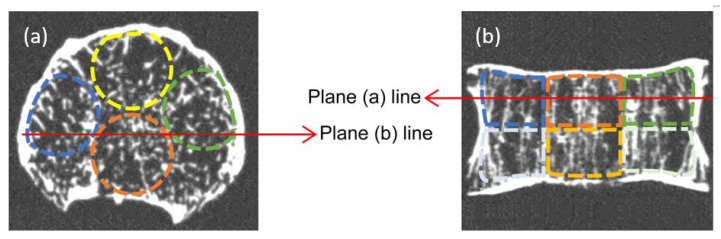
Volume of interest determination in cancellous bone. (**a**) Representative transverse image. (**b**) Coronal image. Both images are obtained by quarter-detector computed tomography. In the transverse direction, there were the following four regions: the ventral and dorsal directions, and the left and right directions. In the coronal image, eight cylindrical regions were set, which were divided into two from the center of the vertebral body in the cranial and caudal directions.

A 3D image analyzer (TRI/3D-BON-FCSCL; Ratoc System Engineering Co., Ltd., Tokyo, Japan) was used to acquire morphometric parameters related to the trabecular bone structure. As random image noise occurs in CT images, gradation processing to convert the images to binary images was performed before carrying out trabecular bone structure measurements. By performing binarization processing, it is possible to remove image noise and extract a bone region. Binarization was performed with a threshold calculated by discriminant analysis between cancellous bone and the bone marrow cavity, using a histogram for the region of interest. After extracting the bone region by binarization processing, volumes of interest (VOIs) to be measured were set. A VOI was set to divide the cancellous bone region of the vertebral body into eight segments. Areas were set up just below the endplates to divide the coronal plane of the vertebral body into two halves in the cranial and caudal directions. Then, transverse VOIs of the vertebral body were defined such that it was divided into four areas in the anteroposterior direction (ventral dorsal direction) and in the lateral direction (Figure 1). Each region was a cylindrical VOI with a diameter of 16 mm and height of approximately 12 mm, and did not include the cortical bone. Figure 1 shows the 3D model and VOIs used for measurement. The morphometric parameters of cancellous bone microarchitecture [28] are shown in Table 1.

### 2.3. Statistical Analysis

JMP ver.14.2.0 (SAS Institute, Cary, NC, USA) was used for statistical processing. Statistical analysis was performed on the relationship between modalities of micro-CT and QDCT images regarding the measured VOIs and average value of all VOIs. Significant differences between micro-CT and QDCT were assessed using the Mann–Whitney test, with respect to the calculated mean measurements. Single regression analysis was performed using a linear regression model with micro-CT as the explanatory variable and QDCT as the objective variable to determine the linear relationship of the measured values between the modalities. An F-test and the coefficient of determination in the linear regression equation were calculated and used. The level of significance was set at *p* < 0.05.

## 3. Results

### 3.1. Morphometric Parameters of Cancellous Bone Microstructure

Table 2 shows the calculation of the morphometric parameters of cancellous bone. The tissue volume (TV) was comparable to the micro-CT values. The bone volume (BV), bone surface (BS), and BV/TV were higher for QDCT. Connectivity density and total skeletal line length did not show significant differences. The degree of trabecular discontinuity was comparable between modalities. In the direct measurements, Tb.Th demonstrated wider values than in QDCT. Regarding Tb.N and Tb.Sp, the measured values were equivalent to those of micro-CT. Concerning the fractal dimension value in QDCT, it was shown that the trabecular bone structure had greater 3D complexity and filled the space compared to micro-CT. The trabecular bone pattern factor (TBPf) and the structure model index (SMI) are bone morphometric parameters that indicate spatial structure. TBPf is <0 when the trabecular bone is honeycomb-like and increases as the trabecular bone acquires a rod-like structure. Concerning the SMI, it approaches values of 3 points as the trabecular bone becomes rod-like. From the results of this study, micro-CT images showed that the trabeculae had a rod-like structure. Marrow space star volume (V*m.space) and trabecular star volume (V*tr) are also quantitative parameters that indicate trabecular continuity. V*m.space increases and V*tr decreases as continuity decreases. QDCT showed increased trabecular continuity relative to micro-CT.

### 3.2. Relationship between Cancellous Bone Morphometric Parameters as Measured by Different Modalities

**Figure 2:**
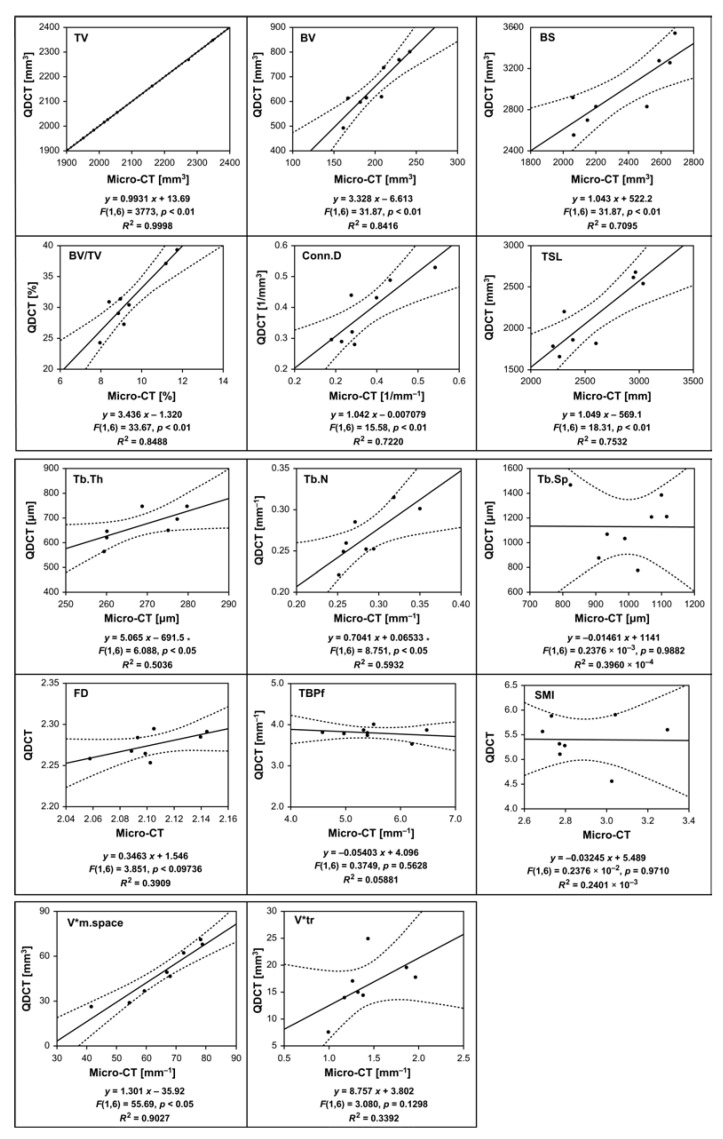
Scatter plot of cancellous bone morphometric parameters of QDCT images against micro-CT images. Solid lines are single regression lines, and dotted lines are 95% confidence intervals for the regression lines. Single regression analyses were performed to calculate the linear regression equations. R2 represents the coefficients of determination. The horizontal and vertical scale intervals may differ.

Figure 2 shows a scatter plot of cancellous bone morphometric parameters. A linear regression model was used with micro-CT as the explanatory variable and QDCT as the objective variable. No significance was observed for Tb.Sp, FD, TBPf, SMI, and V*tr for the regression equation using an F-test. A significant linear regression model could be applied for other bone morphometric parameters. All the bone morphometric parameters to which the linear regression equation could be applied resulted in positive coefficient estimates for explanatory variables.

### 3.3. Characteristics of Cancellous Bone Images

**Figure 3:**
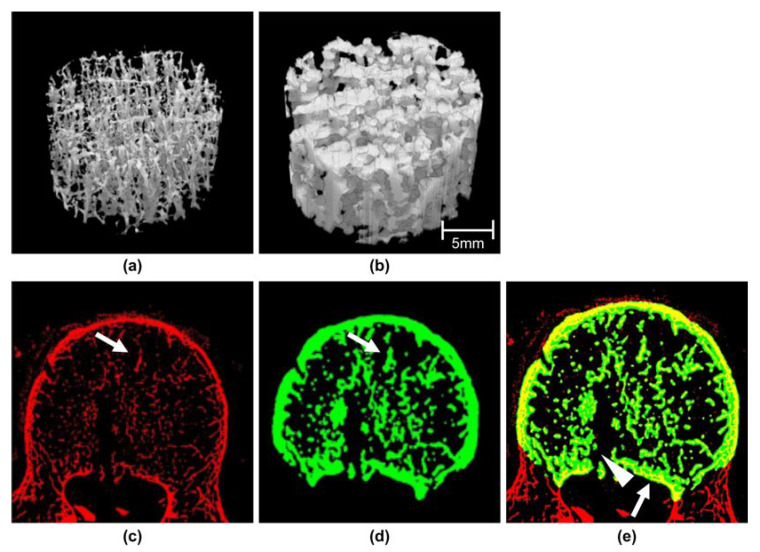
Cancellous bone images from micro-computed tomography (micro-CT) and QDCT. (**a**) Three-dimensional (3D) image of the cancellous bone obtained by micro-CT. (**b**) Similar to (**a**) but obtained by QDCT. The VOI used for the 3D image display was set behind the upper part of the cancellous bone of the lumbar vertebrae. (**c**) Transverse image of the lumbar spine obtained by micro-CT. (**d**) Transverse image obtained by QDCT. (**e**) Fusion image of lumbar spine transverse images obtained by micro- and QDCT.

Figure 3 shows 3D and 2D images of the cancellous bone. There are visual differences between the modalities when compared in the 3D images. The cancellous bone extracted from micro-CT is thin (Figure 3c arrow), whereas the cancellous bone at the same site (Figure 3d arrow) extracted from QDCT is several times thicker. In the fusion image (Figure 3e), overlapping regions are displayed in fusion colors. In many cancellous bone regions, micro-CT images are superimposed on QDCT images. The site where the cancellous bone gathers (arrowhead in Figure 3e) is shown without being resolved in the QDCT image. In QDCT, as the bone width increases, agreement with micro-CT increases (Figure 3e arrow).

## 4. Discussion

In this study, we evaluated the relevance of measurements to micro-CT for trabecular structure determination using QDCT. We investigated the 3D micromorphology used to evaluate bone fragility in the cancellous bone of the specimen lumbar vertebrae. The QDCT scanner used here can acquire images with high spatial resolution, as compared to the currently used MDCT scanners for the whole body. Thus, we evaluated the relatedness between QDCT and micro-CT in the analysis of the trabecular structure of lumbar cancellous bone. In previous studies, micro-CT images were often used as a reference [28,36,37]. The micro-CT scanner is used for industrial non-destructive examination and can acquire images with significantly higher spatial resolution than a medical CT scanner. When considering using QDCT that has been put into practical use for the analysis of trabecular structure, where spatial resolution affects the measurement results, it is reasonable to consider its relationship to micro-CT.

As presented in Figure 1 and Figure 2, there was no difference in the TV or cancellous bone surface measurements. In addition, the regression coefficient for the measured values in micro-CT was close to 1; the same results may be obtained by QDCT. Meanwhile, the fact that the regression coefficient for the cancellous bone volume is higher than that for the bone surface is likely attributed to the spatial resolution of QDCT. A previous study also addressed the spatial resolution of MDCT images [38]. As shown in Figure 3, the fact that the site where the trabeculae aggregated tends to be imaged as a mass is presumed to lead to higher volumes being measured by QDCT. Since the linearity of the measured values between modalities has been shown, results similar to those reported for micro-CT can be obtained by correcting the QDCT. BV/TV is related to bone mineral density and is an important bone morphometric parameter for assessing bone fragility. The accuracy of cancellous bone volume measurements may account for the regression coefficient of BV/TV being greater than 1. Micro-CT has a spatial resolution of 0.05 mm and has isotropic voxels. The QDCT spatial resolution is estimated to be ≤0.25 mm, as it depends on slice thickness. Human cancellous bone is 0.1–0.3 mm thick. According to the Nyquist–Shannon sampling theorem, measuring trabecular thickness requires a spatial resolution of ≤0.05 mm. The regression coefficient difference in Figure 2 indicates a difference in the spatial resolution. Nevertheless, the association between modalities was recognized. Therefore, by correcting the measured values using appropriate coefficients, it is presumed that even if QDCT is used, similar values to those reported for micro-CT can be obtained.

Connectivity density and the total skeletal line length, which are related to trabecular connectivity, yield linear relationships between modalities. The total skeletal line length is measured at the center of cancellous bone and is not affected by cancellous width. As a result, calculating accurate values is possible even with QDCT. Considering the relationship with BV/TV, trabecular bone volume can be calculated by the product of the total skeletal line length and the trabecular bone. In our study, the regression coefficient of BV/TV was similar to that of BS and consistent with the BV calculation method. Our results indicated improved measurement precision compared to conventional MDCT [39]. QDCT can serve as evidence for adequate spatial resolution to obtain measurement values equivalent to those obtained for trabecular structure analysis using micro-CT. In comparison with previous MDCT studies [38,40], BV/TV was approximately double that of micro-CT. Our findings indicate that the validity of the trabecular structure analysis is higher than that of conventional MDCT.

Tb.Th, Tb.N, and Tb.Sp constitute direct measurements of trabecular morphology, suggesting that they are susceptible to spatial resolution. Tb.Th and Tb.N were used as regression coefficients, showing the relationship between the measured values. As for Tb.Th, QDCT was found to be expanded approximately five times. Figure 3 also shows features of the QDCT images. A linear relationship was not obtained for Tb.Sp, and the influence of spatial resolution appears to have been eliminated because there is no difference in the average measured values between modalities. Additionally, Tb.Th and Tb.N measurements required corrections according to the spatial resolution in our study when judging bone fragility by trabecular structure analysis. Regarding Tb.Sp, although the measured value from QDCT could be used, it has been reported to be contradictory to Tb.Th [15,41]. We can hypothesize that there was sufficient space for spatial resolution in terms of Tb.Sp, as there was no difference in the average measured value between the modalities. When measuring cancellous bone with a denser trabecular bone structure than the one we used for our specimen, the result may be reversed. Thus, it is necessary to examine each value according to the trabecular bone structure.

Based on the results of BV/TV and Tb.Th, to evaluate even finer structures, QDCT requires several times higher spatial resolution. We suggest that the values of some cancellous bone morphometric parameters obtained by QDCT were insufficiently accurate and that a measurement method with higher spatial resolution was required. A larger fractal dimension may result in a higher occupancy rate of the trabecular bone structure in the tissue, which is a texture index that represents the state of healthy bones. Osteoporotic cases tend to have reduced trabecular complexity [42]. Regarding the fractal dimension, no linear relationship was observed between the modalities, but there was a significant difference in the measured values, and the QDCT-measured values were high. It was suggested that QDCT is inferior to micro-CT in its ability to display 3D trabecular structures. According to the fractal dimension, the accuracy of the measured value obtained by QDCT is low, and there is a possibility of underestimating bone fragility. TBPf and SMI did not show a linear relationship, despite the fact that there were significant differences in the mean measurements. Concerning the analysis of SMI using MDCT, its correlation with micro-CT was reported to be small in a previous study [43]. These bone morphometric parameters are related to the mean curvature of the trabecular surface [44]. By using the morphometric parameters of the trabecular bone structure, it is possible to quantitatively evaluate whether the trabecular bone is a plate- or rod-like structure. From the micro-CT measurements, we inferred that the cancellous bone used in this study contained an extensive rod-like structure. QDCT analysis of TBPf showed a plate-like structure. Regarding the spatial structure of trabecular bone, plates are more resistant to fracture than rods. Therefore, as with the fractal dimension, there is a possibility of overestimating bone strength when evaluating fractures.

V*m.space and V*tr are the same as Tb.Th and the other coefficients described above in that they use direct measurements, but differ in the distance calculation algorithm. As V*m.space measures the bone marrow space surrounding the trabecular bone, QDCT spatial resolution had little effect, and a linear relationship with micro-CT was noted. The regression coefficient of V*tr was greatly affected by spatial resolution compared to V*m.space. This is attributed to the orthogonal direction of the cancellous bone. Moreover, the extension direction was affected by the volume calculation. The influence of the region where the cancellous bones swelled and aggregated, as shown in Figure 3, was evident. To verify the validity of the measured values in trabecular bone structure analysis, it is important to understand the image characteristics of the scanner used and the method of calculating bone morphometric parameters.

Trabecular structure analysis by QDCT can be clinically useful in assessing bone fragility, regardless of sex. It can be applied to cases of hormone therapy and for the evaluation of osteoporosis in young people, including cases of secondary osteoporosis due to lifestyle-related diseases. The benefit of clinical diagnosis should consider the risks from radiation exposure for young people.

Notably, the specimen used for QDCT in this study was not directly obtained from a living body. First, some information regarding the original lumbar cancellous bone mineral density may have been lost when preparing the 3D model. Second, in vivo, the intertrabecular tissue is the bone marrow, but this aspect is not reproduced in a 3D model. In this study, binarization processing was performed to extract the trabecular bone as in actual trabecular structure analysis, although changes in trabecular extraction due to the presence of bone marrow have not been considered. This may have caused the noise in the image to affect the binarization process [45]. In CT scans, it is common to reduce the imaging dose to reduce the effects of radiation exposure on the human body. In this study, the effect of image noise was eliminated by setting a sufficient imaging dose; however, clinically, image noise may affect bone trabecular extraction. Third, it should also be mentioned that the material used is also not living bone (containing calcium phosphate). Bone densitometry using CT values was not performed because resin for creating a 3D model was used. In bone fragility evaluation, measurements of bone mineral density and bone microstructure are performed, but for the aforementioned reasons, this evaluation was limited to trabecular bone morphological structure analysis. Further consideration of these aspects, combined with bone mineral density evaluation, is also necessary.

## 5. Conclusions

We conclude that by correcting the measured values, it is possible to perform an analysis equivalent to micro-CT, which offers higher spatial resolution than QDCT.

We found evidence that if QDCT could be used, trabecular structure evaluation could provide additional information. In addition to image diagnosis, highly accurate quantitative analyses are possible by combining the trabecular structure analysis with the bone mineral densitometry that is performed in examinations using conventional MDCT. QDCT still does not have sufficient spatial resolution for analysis of the trabecular structure of cancellous bone. Nevertheless, by applying necessary corrections to micro-CT, it is possible to use it for bone fragility assessment.

## Figures and Tables

**Table 1:** Description of 3D parameter for cancellous trabecular bone microarchitecture.

Bone Morphometric Parameters (Abbreviations)	Description
Tissue volume (TV)	The volume, including the bone marrow cavity near the cancellous bone; bone tissue volume
Bone volume (BV)	Volume of the trabecular cancellous bone
Bone surface (BS)	Surface area of the trabecular cancellous bone
Bone volume fraction (BV/TV)	Volume of the cancellous bone relative to the bone marrow cavity; trabecular bone volume fraction
Connectivity density (Conn.D) [29,30]	Connectivity density of trabecular bone structure; when b is the number of branches of the trabecular bone structure, it is calculated as b/TV
Total strut length (TSL) [29]	The total distance of the skeletal network; represented by classifying trabeculae into connection points between the trabeculae, end points that are not connected with other trabeculae, and connection points with the cortical bone
Trabecular thickness (Tb.Th) [29,31]	Trabecular bone width; the distance directly measured to the trabecular surface within the trabecular bone; the diameter of the sphere contained in the trabecular bone is twice the resulting value; trabecular thickness is the average maximum diameter of these spheres
Trabecular number (Tb.N) [29,31]	Number of trabecular bones; it represents the number of trabeculae per unit length that crosses a straight line perpendicular to the longitudinal direction of the trabecular bone
Trabecular separation (Tb.Sp) [29,31]	Trabecular spacing; the distance to the trabecular surface is directly measured within the bone marrow region; the diameter of the spheres contained in the bone marrow is twice the resulting value; trabecular spacing is the mean maximum diameter of these spheres
Fractal dimension (FD) [29,32]	Fractal dimensionality; it represents the complexity of the unevenness of the trabecular surface
Trabecular bone pattern factor (TBPf) [29,33]	A quantitative index of the three-dimensional concave structure; it represents the change in surface area relative to the change in near-surface volume of trabecular bone; this value is 0 in plate-like structures
Structure model index (SMI) [29,34]	An index of the three-dimensional morphology of trabecular bone; 0, 3, and 4 points indicate ideal plate-like, rod-like, and sphere-like structures, respectively
Marrow space star volume (V*m.space) [29,35]	An index of continuity in the bone marrow cavity based on the star-line algorithm; it is the average volume of the marrow cavity that can be traced from a central point in the marrow cavity in all directions
Trabecular star volume (V*tr) [29,35]	An index of continuity in trabecular bone based on the star-line algorithm; it is the average volume from a central point in the trabecular bone to the trabecular edge in all directions.

**Table 2:** Mean values of morphometric parameters in the cancellous bone.

Bone Morphometric Parameters	Micro-CT	QDCT	*p*-Value
TV (mm^3^)	2102 ± 135	2102 ± 134	1.00
BV (mm^3^)	198.9 ± 26.9	655.2 ± 97.5	*
BS (mm^2^)	2364 ± 254	2988 ± 314	*
BV/TV (%)	9.470 ± 1.237	31.22 ± 4.61	*
Conn.D (1/mm^3^)	0.3752 ± 0.0758	0.3839 ± 0.0929	0.875
TSL (mm)	2590 ± 324	2147 ± 392	0.0611
Tb.Th (μm)	267.6 ± 8.2	663.8 ± 58.3	*
Tb.N (1/mm)	0.2860 ± 0.0317	0.2667 ± 0.0290	0.270
Tb.Sp (μm)	996.1 ± 95.1	1126.5 ± 220.7	0.270
Fractal dimension	2.104 ± 0.026	2.275 ± 0. 014	*
TBPf (1/mm)	5.483 ± 0.573	3.800 ± 0.128	*
SMI	2.890 ± 0.197	5.395 ± 0.413	*
V*m.space (mm^3^)	64.91 ± 11.82	48.51 ± 16.18	0.0661
V*tr (mm^3^)	1.425 ± 0.311	16.28 ± 4.67	*

*, *p* < 0.01. Values are indicated as means ± standard deviations.

## Data Availability

The data are available from the corresponding author upon reasonable request.
